# Bone and joint tuberculosis: clinical manifestation, diagnostic techniques and drug resistance analysis

**DOI:** 10.3389/fcimb.2025.1687906

**Published:** 2025-11-21

**Authors:** Yingying Yuan, Pengxiang Li, Xiangjie Qiu, Honghua Zhang, Hao Chen, Nan Zang, Wenbo Li, Meijin Cheng, Zhen Guo, Xiaodong Niu, Yue Zhao, Xiuli Cao, Yungang Han, Wei Wang

**Affiliations:** 1 Department of Clinical Laboratory, Henan Provincial Chest Hospital, Zhengzhou University, Zhengzhou, China; 2 Henan Provincial Key Laboratory of Tuberculosis Diagnostic Medicine, Zhengzhou, China; 3 Henan Provincial Infectious Diseases (Tuberculosis) Clinical Medical Research Center, Zhengzhou, China; 4 Cardiac Surgery Intensive Care Unit, Henan Provincial Chest Hospital, Zhengzhou University, Zhengzhou, China; 5 Department of thoracic surgery, Henan Provincial Chest Hospital, Zhengzhou University, Zhengzhou, China

**Keywords:** bone & joint tuberculosis, drug resistance, GeneXpert MTB/RIF, *Mycobacterium tuberculosis*, osteoarticular tuberculosis

## Abstract

**Objectives:**

Clinical data on bone and joint tuberculosis (BJTB) in developing countries remain limited. This study aims to investigate the clinical, epidemiological, and drug-resistance characteristics of BJTB patients in Central China and to optimize diagnostic strategies.

**Methods:**

This retrospective study analyzed data from patients diagnosed with BJTB at Henan Provincial Chest Hospital between 2016 and 2022.

**Results:**

Among the 902 patients, 518 (57.4%) were male and 384 (42.6%) were female. The age groups of 21–30 years and 51–60 years, as well as the rural population, showed the highest prevalence of cases. Local pain was the most prevalent symptom, followed by fever, night sweats, and neurological dysfunction. On average, the time from symptom onset to diagnosis was 6.1 months, and the mean hospital stay was 64.2 days. Spinal tuberculosis was the most frequently affected site, accounting for 77.6% (700/902) of cases. Five diagnostic techniques were evaluated, with GeneXpert MTB/RIF demonstrating superior performance by achieving a sensitivity of 91.6% (95%CI: 86.3%-95.0%) and specificity of 90.1% (95%CI: 85.5%-93.6%). Unlike drug resistance patterns observed in other regions, streptomycin (29.6%) and rifabutin (18.2%) were the most frequently encountered first-line and second-line anti-tuberculosis drugs, respectively. The prevalence of multidrug-resistant tuberculosis (MDR-TB) was 8.3% (95% CI: 5.1%–13.2%), and extensively drug-resistant tuberculosis (XDR-TB) was identified in 1.6% (95% CI: 0.2%-5.6%) of cases.

**Conclusions:**

The application of GeneXpert MTB/RIF demonstrated significant diagnostic accuracy for BJTB. The control of MDR-TB remains a critical challenge in the management of BJTB in Central China.

## 1 Introduction

To date, tuberculosis (TB) remains a significant infectious disease that poses a grave threat to human health, and it has caused almost twice as many deaths as HIV/AIDS (Organization WH and World Health Statistics, 2024). Until the COVID-19 pandemic, TB was the leading cause of death from a single infectious agent (Kumar et al., 2024). Although the global incidence of TB has decreased in recent years, the decline has been far less than targeted in the World Health Organization’s “End the TB Epidemic” strategy. In 2024, the World Health Organization (WHO) reported a global total of 10.8 million new cases of TB and 1.25 million TB-related deaths. China has 741,000 new cases, ranking 3rd out of 30 high TB burden countries after India and Indonesia (Organization WH, 2024).

TB predominantly affects the lungs and is predominantly transmitted via the respiratory tract, thus causing pulmonary tuberculosis (PTB). However, in cases of compromised immune function, *Mycobacterium tuberculosis* (Mtb) may disseminate through the lymphatic system or bloodstream, leading to extrapulmonary tuberculosis (EPTB). In China, the incidence of EPTB is approximately 24.6%. The commonly affected sites include the pleura (35%), musculoskeletal system (15.8%), lymphatic system (15.8%), gastrointestinal tract (8.1%), genitourinary organs (7.8%), and central nervous system (1%), among others. Due to atypical clinical presentations, challenges in specimen collection, and low bacterial load, the diagnosis of EPTB remains clinically difficult (Li T. et al., 2023).

Bone and joint tuberculosis (BJTB), one of the most prevalent forms of EPTB, accounts for approximately 11.3% of all TB cases, with its incidence among EPTB cases ranging from 10% to 21.1% (Dunn and Ben Husien, 2018; Khan et al., 2021). BJTB is thought to result from hematogenous dissemination and reactivation of dormant bacilli during episodes of bacteremia, often following primary infection (Khan et al., 2022). The spine and weight-bearing large joints—particularly the hip and knee—are most commonly involved. Due to its insidious onset and overlapping clinical and radiological features with various inflammatory arthritides, such as rheumatoid arthritis, gout, crystal-induced arthropathies, and ankylosing spondylitis, the diagnosis of BJTB remains challenging and often delayed. Most patients do not simultaneously present active PTB, especially those with multiple vertebral lesions, often resulting in severe dysfunction and even paraplegia, which seriously affects the patients’ lives (Procopie et al., 2017; Kamal et al., 2022). Early diagnosis and treatment are of great significance for the management of BJTB.

The prevalence of TB is determined by factors such as geographical location, lifestyle, socioeconomic status, and access to medical services (Duarte et al., 2018). Currently, BJTB is not classified as a notifiable infectious disease in China, hence its precise incidence and drug resistances rates remains unknown. A comprehensive report detailing BJTB is evidently lacking. To date, only a limited number of studies have been conducted on BJTB patients in southwest China (Shi et al., 2016; Liu et al., 2019; Zeng et al., 2021; Qin et al., 2024). Henan Province, located in central China, is not only the most populous region but also the fourth province with the highest burden of TB, featuring a prevalence of 41,732 (Center PHD, [[NoYear]]). Therefore, more attention needs to be paid to the prevention and treatment of TB in this province. Nevertheless, no study focuses on the prevalence, clinical characteristics or drug resistance characteristics of BJTB in central China to date. The current retrospective analysis encompasses 902 cases of BJTB admissions at Henan Provincial Chest Hospital from January 2016 to December 2022. It aims to raise greater awareness regarding bone extension, to enhance our understanding of its clinical data, drug resistance profiles, diagnosis and management, and to provide guidance for individualized treatment strategies and grassroots promotion.

## 2 Materials and methods

### 2.1 Patients survey

This study was conducted at Henan Province Chest Hospital, a tertiary medical center specializing in the diagnosis and treatment of TB in Henan Province. We retrospectively reviewed the medical data of patients who were admitted to the Department of Orthopedics and TB at the study hospital for BJTB during the period 2016 -2022. This study was approved by the hospital Ethics Committee and informed consent was obtained from all patients.

### 2.2 Case definition and data collection

We collected patient data, including demographics, medical histories, clinical presentations, imaging findings, histopathological analyses, laboratory tests, anti-TB treatment regimens, and prognosis. Cases included in our analysis met the following criteria: (1) confirmed BJTB patients hospitalized at our institution, (2) radiologic findings, pathological or etiology diagnosis results meeting diagnostic criteria for bone TB, and (3) Empirical anti-TB chemotherapy was effective when it was difficult to obtain a definite diagnosis. Patients with incomplete course records and inadequate examination data were excluded.

### 2.3 Comparison of five inspection techniques

Participants in this study were asked to provide specimens resected during operation and blood for examination, including smear, culture, GeneXpert MTB/RIF, TB-DNA, T-SPOT.TB, and TB-IgG antibody testing. 0.1ml of the treated sample was inoculated onto a modified Roche medium according to the simple method of the Laboratory Test Protocol for the Diagnosis of TB. The medium was cultured in a constant temperature incubator at 37°C. The culture was observed on the third and seventh days after inoculation, and then the colony growth was observed once a week. The culture was positive if the colony was found to grow and was positive after acid-fast staining. The specimens were ground and mixed with sample reagent at a ratio of 1:2, then incubated for 20–30 minutes and detected by the test system GeneXpert^®^ Infnity-48 s. (Cepheid, Sunnyvale, CA). According to the Ct value, Mycobacterium TB load was calculated. For TB-DNA, the bacterial load is detected according to the manufacturer’s instructions (Xiamen Zhishan Biotechnology Co., LTD). For T-SPOT.TB assay, whole blood samples were collected by heparin anticoagulant and incubated at 37°C. Peripheral blood mononuclear cells were then isolated from blood samples, collected and counted according to the manufacturer’s instructions (Shanghai Fosun Diagnostic Technology Co., LTD.). It stimulates peripheral blood mononuclear cells using Mtb specific antigens, namely ESAT-6 (Rv3875) and CFP-10 (Rv3874), and subsequently detects the response of antigen-specific T lymphocytes by measuring the secretion of the cytokine interferon-γ. For the TB-IgG antibody assay, the sera samples were used to detect the IgG antibodies against Mtb by the respective manufacturer’s instructions (Shandong Kanghua Bio-Medical Technology Co. LTD). Detection performance indicators including sensitivity, specificity, positive predictive value, negative predictive value, and the Youden index (YI) were used to evaluate performance.

### 2.4 Drug susceptibility testing

The Mtb H37Rv strain was utilized as the control sample. Drug susceptibility testing was performed using the absolute concentration method recommended by the WHO, and the concentrations of drugs were as follows: isoniazid (INH), 0.2 μg/mL; rifampicin (RFP), 40.0 μg/mL; streptomycin (SM), 4.0 μg/mL; ethambutol (EMB), 2.0 μg/mL; rifabutin (RBU), 20 μg/mL; kanamycin (KAN), 30.0 μg/mL; levofloxacin (LFX), 2.0 μg/mL; moxifloxacin (MFX), 1.0 μg/mL; capreomycin (CPM), 40.0 μg/mL; amikacin (AK), 30.0 μg/mL; prothioconazole (TH), 40.0 μg/mL; p-aminosalicylic acid (PAS), 1.0 μg/ml.

### 2.5 Evaluation of clinical outcomes

The prognostic information of the study population was acquired via medical records, telephone, or outpatient follow-up visits. Clinical manifestations, computed tomography (CT), and magnetic resonance imaging (MRI) were recorded before surgery, 1 month, 3 months after surgery, and every 6 months thereafter. The criterion for achieving cure includes the following: after a 2-year treatment period, maintenance of ESR and CRP within the normal range, absence of TB lesion expansion in imaging examinations, observation of bone healing at the lesion site, and disappearance of clinical symptoms for 3 months.

### 2.6 Statistical analysis

Quantitative data were depicted as mean ± SD, while qualitative data were presented in numbers and percentages. Analysis and mapping were created with the GraphPad Prism 8.0 and Microsoft Excel 2016 software. Other statistical analyses were performed with the SPSS 21.0 software (IBM Corporation, Armonk, NY, United States). The chi-square test was used for classification analysis, while the ANOVA test was applied for the comparison of continuous data. The 95% confidence interval (95% CI) for the drug resistance rate was calculated using the Wilson Score method; however, the Clopper-Pearson method was applied in cases of small sample sizes to ensure greater accuracy. P value less than 0.05 was considered statistically significant.

## 3 Results

### 3.1 Epidemiological characteristics

**Figure 1:**
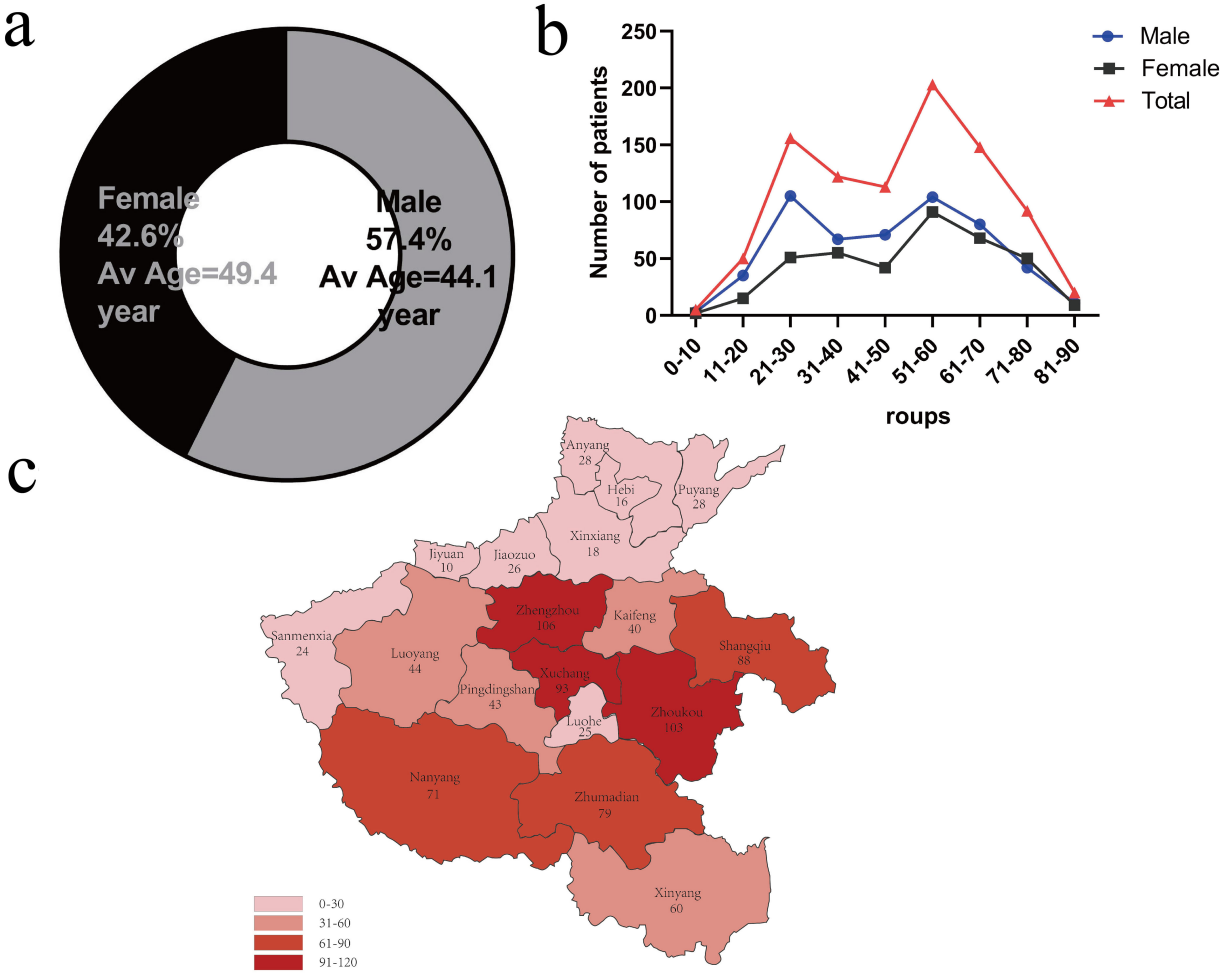
Demographic characteristics of BJTB cases in central China. **(a)** The sex and average age of the cases; **(b)** The distribution of the cases according to age; **(c)** The regional distribution of the cases.

From 2016 to 2022, our hospital managed a total of 38,592 patients diagnosed with TB. Of these, 29,214 cases were PTB, representing 75.7% of the total, while 9,378 cases were EPTB, accounting for 24.3%. Among the EPTB cases, BJTB was one of the most common subtypes, comprising 1,342 cases (14.3%). Other forms of EPTB included pleural-TB (3,545 cases, 37.8%), lymph-node TB (1,294 cases, 13.8%), gastrointestinal-TB (1,003 cases, 10.7%), peritoneal-TB (900 cases, 9.6%), genitourinary-TB (703 cases, 7.5%), meningitis-TB (131 cases, 1.4%), and TB at other sites (460 cases, 4.9%). During the 7-year period, the incidence of BJTB was relatively stable (169–225 new cases per year). Of the 902 participants, 518 (57.4%) were male with a mean age of (44.1 ± 13.1) years, and 384 (42.6%) were female with a mean age of (49.4 ± 18.6) years, resulting in a male-to-female ratio of 1.4:1. (**Table 1**, **Figure 1a**). The age distribution exhibits a bimodal pattern, with the highest concentrations observed in the 21–30 and 51–60 age groups (**Table 1**, **Figure 1b**). The incidence of BJTB was higher in the rural population (73.3%) than in the urban population (26.7%, P < 0.05). No difference was found between spinal TB and joint TB patients in gender, and age distribution (P > 0.05). Statistical data regarding regional distribution indicate a relatively higher incidence of BJTB in the central and southeastern cities of Henan Province. Geographically, the majority of patients were from Zhengzhou City (11.8%, 106/902), followed by Zhoukou City (11.4%, 103/902), Xuchang City (10.3%, 93/902) and Shangqiu City (9.8%, 88/902) (**Figure 1c**).

**Table 1:** Demographic characteristics of 902 patients with BJTB.

Characteristic	Total (n=902)	Spinal TB (n=700)	Joint TB (n=189)	Other bone TB (n=13)	P
Sex					
Male, No. (%)	518 (57.4)	412 (58.9)	98 (51.9)	8(61.5)	0.21
Female, No. (%)	384 (42.6)	288 (41.1)	91 (48.1)	5(38.5)	
Age, mean (range), year	48.1 (5–94)	48.5 (5–94)	46.6 (10–86)	46.1 (21–76)	0.44
Age, distribution, No. (%)					
0-10	5 (0.6)	4 (0.6)	1 (0.5)	0 (0.0)	
11-20	51 (5.6)	39 (5.6)	12 (6.3)	0 (0.0)	
21-30	158 (17.5)	121 (17.3)	34 (18.1)	3 (23.1)	
31-40	122 (13.5)	94 (13.4)	26 (13.8)	2 (15.4)	
41-50	113 (12.5)	82 (11.7)	29 (15.4)	2 (15.4)	
51-60	195 (21.6)	148 (21.1)	43 (22.9)	4 (30.8)	
61-70	146 (16.5)	119 (17)	26 (13.8)	1 (7.7)	
71-80	92 (10.2)	74 (10.6)	17 (9.1)	1 (7.7)	
81-90	20 (2.2)	19 (2.7)	1 (0.5)	0	
Rural population, No. (%)	661 (73.3)	533 (76.1)	119 (62.8)	9 (69.2)	<0.01
Urban population, No. (%)	241 (26.7)	167 (23.9)	70 (37.2)	4 (30.8)	
Basic disease, No. (%)	327 (36.3)	263 (37.6)	69 (36.5)	2 (15.4)	0.26
Hypertension	136 (15.1)	104 (14.9)	31 (38.6)	1	
Diabetes mellitus	74 (8.2)	58 (3.6)	15 (7.9)	1	
Hepatitis B	41 (4.5)	29 (4.1)	12 (6.3)	0	
Immunosuppression	43 (4.8)	27 (3.9)	16 (8.5)	0	
Cardiovascular disease	29 (3.2)	25 (3.6)	4 (2.1)	0	
cerebral embolism	17 (1.9)	17 (2.4)	0 (0.0)	0	
Others	39 (4.3)	34 (3.4)	5 (2.6)	0	

### 3.2 Clinical presentations

**Figure 2:**
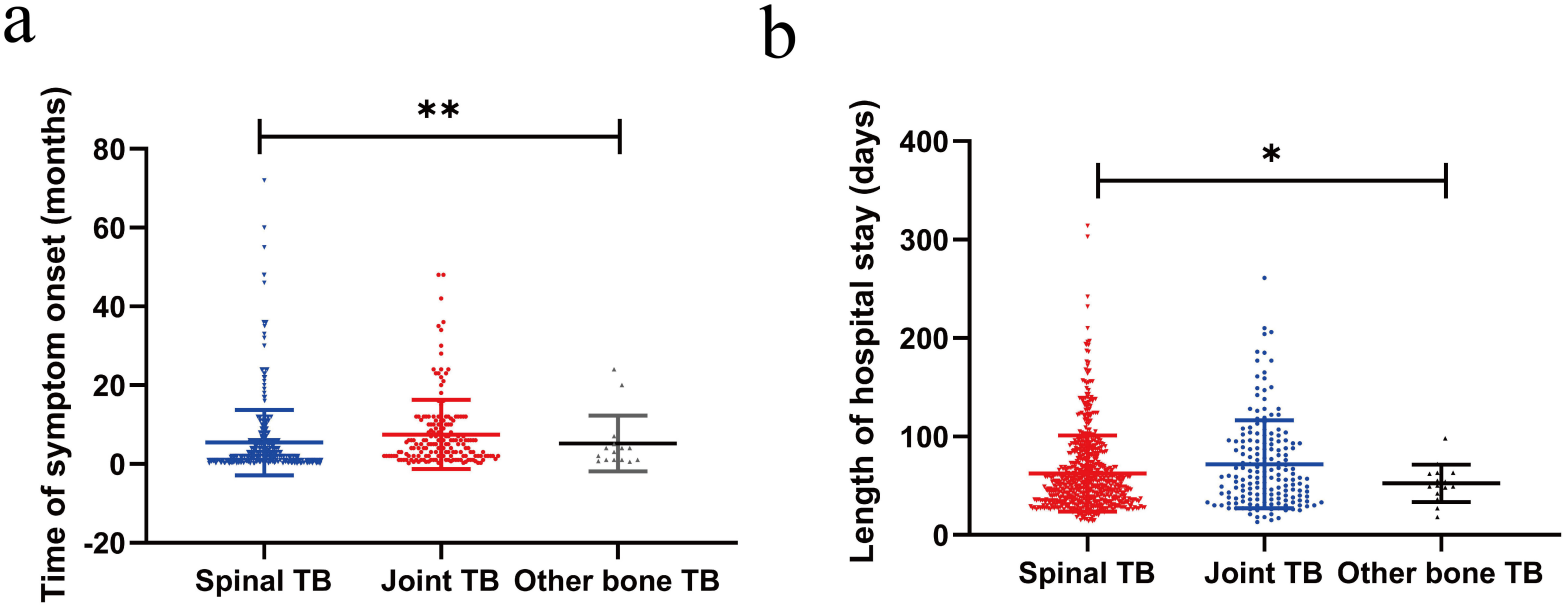
Comparison of clinical manifestations and duration of therapy for spinal TB and joint TB **(a)** The time of symptom onset; **(b)** The length of hospital stay. *p<0.05; **p<0.01.

Overall mean duration from symptom onset to diagnosis was 6.1 months (range, 0.1–72 months), and there was a significant difference in the duration of time to diagnosis of the spinal patients (5.4 months), the joint patients (7.3 months) and other bone TB patients(7.3 months, P <0.05, **Table 2**, **Figure 2a**). A total of 105 (11.6%) patients have a history of TB, and 9 (1.0%) patients have a history of fracture operation. Local pain was the most common symptom (808, 89.6%), followed by fever (290, 32.2%, neurological dysfunction (223, 24.7%), night sweats (165, 18.3%), weight loss (≥5 kg,162, 18.0%), local mass (96, 10.6%), and swelling (40, 4.4%). Patients with spinal TB had a higher incidence of local pain (636, 90.9%), fever (239, 34.1%), and night sweats (143, 20.4%) compared to joint TB patients (P <0.05, **Table 2**). In contrast, local mass (45, 23.9%) and swelling (38, 20.1%) were more common in joint TB cases compared to spinal TB (P <0.05, **Table 2**).

**Table 2:** Clinical presentations.

Characteristic	Total (n=902)	Spinal TB (=700)	Joint TB (=189)	Other bone TB (n=13)	P
Time to diagnosis mean (range), month	6.1(0.1-72)	5.4 (0.1-72)	7.3 (0.2-48)	7.3(0.5-26)	<0.05
History of TB, No. (%)	105 (11.6)	80 (11.4)	23 (12.2)	2 (15.3)	0.88
Operation history, No. (%)	9 (1.0)	7 (1.0)	2 (1.1)	0 (0.0)	0.14
Symptoms/signs, No. (%)					
Local pain, No. (%)	808 (89.6)	636 (90.9)	161 (85.2)	11(84.6)	<0.01
Fever, No. (%)	290 (32.2)	239 (34.1)	48 (25.4)	3 (23.1)	0.02
Night sweat, No. (%)	165 (18.3)	143 (20.4)	20 (10.6)	2 (15.3)	0.01
Weight loss (≥5kg), No. (%)	162 (18.0)	136 (19.4)	24 (12.7)	2 (15.4)	0.10
Local mass, No. (%)	96 (10.6)	48 (6.9)	45 (23.9)	3 (23.1)	<0.01
Swelling, No. (%)	40 (4.4)	0 (0.0)	38 (20.1)	2 (15.4)	<0.01
Neurological dysfunction, No. (%)	223 (24.7)	173 (24.7)	49 (25.9)	1 (7.7)	0.45
Pulmonary involvement, No. (%)	310 (34.4)	245 (35.0)	61 (32.3)	4 (30.8)	0.75
Other sites involvement, No. (%)	109 (12.1)	90 (12.9)	18 (9.5)	1 (7.7)	0.41
Hematogenous dissemination, No. (%)	20 (2.2)	16 (2.3)	4 (2.1)	0 (0.0)	0.49
Length of hospital stay mean (range), days	64.2 (13–261)	62.5 (14–314)	70.3 (13–261)	67.8 (13–125)	<0.05

In this study, multifocal TB was present in 329 (37.0%) of 902 patients with BJTB. The most common site of extra-bone involvement was the lung. Concomitant pulmonary involvement was diagnosed in 310 (34.4%) of cases. The overall average length of hospital stay was 64.2 days, and the joint TB patients (70.3 days) stayed longer in the hospital than the spinal TB patients (62.5 days, P <0.05, **Figure 2b**).

MRI was performed in 85.6% of patients and CT scan was performed in 31.8% of patients. Among the spinal TB patients, the lumbar spine was the most commonly involved site (408, 58.3%), followed by the thoracic spine (377, 53.9%). The cervical spine (37, 5.3%) and the sacral spine (69, 9.9%) were less commonly involved. For the joint and other bone TB patients, the hip (44, 21.8%) and knee (43, 21.3%) were the most commonly involved sites, followed by sacroiliac (24, 11.9%), ankle (21, 10.4%), and elbow (18, 8.9%). Digital (5, 2.5%), rib (11, 5.4%), and limbs (2, 1.0%) were less commonly involved.

### 3.3 Value of five techniques in the diagnosis of BJTB

We performed statistics on the hematological data and found that 38.6% (348/902) of the patients had anemia, while 44.4% (401/902) exhibited hypoalbuminemia. Additionally, 80.9% (488/603) of the patients showed elevated levels of C-reactive protein, and 72.1% (555/769) had an accelerated erythrocyte sedimentation rate. No patient was found to be HIV positive.

**Figure 3:**
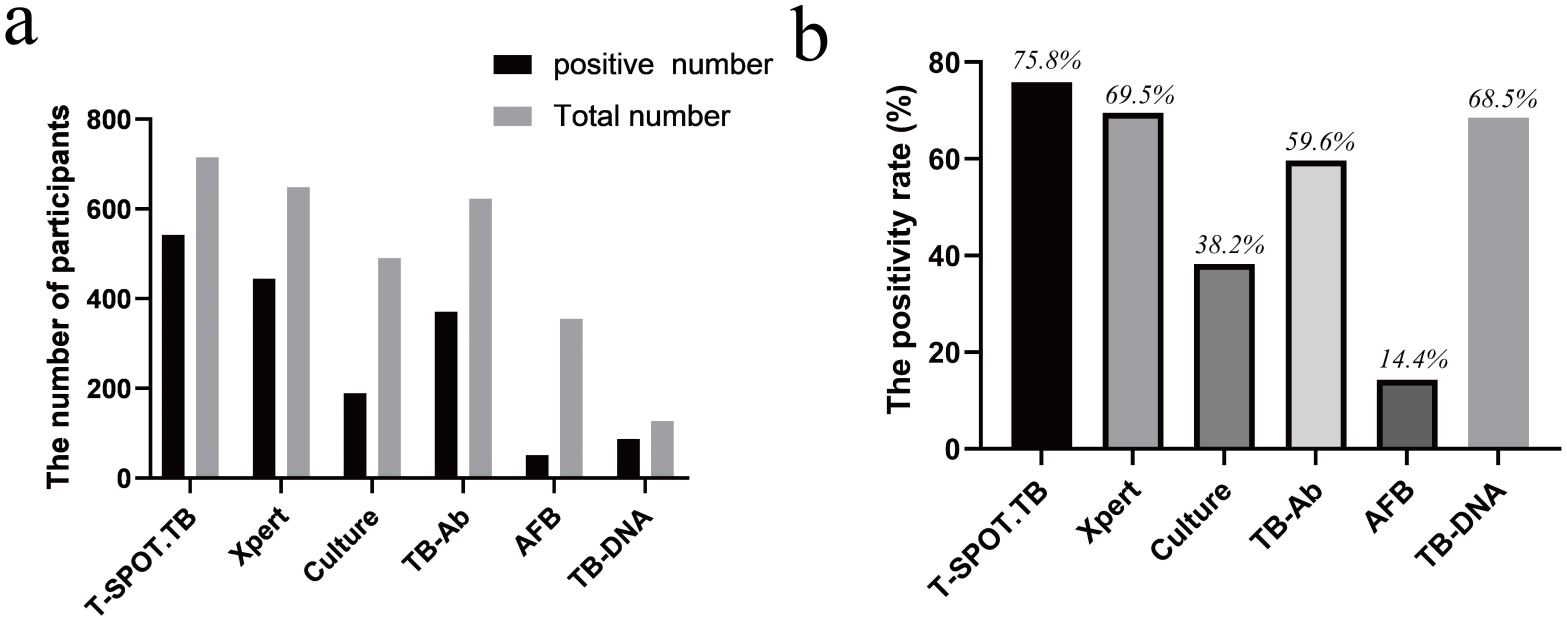
Evaluation of five diagnostic techniques using TB culture as the reference standard. **(a)** The number of participants for five tests; **(b)** The positivity rate percentage for five tests.

Furthermore, 75.8% (542/715) of patients with BJTB tested positive in the T-cell-based tuberculosis test (T-SPOT.TB), while 68.5% (87/127) tested positive in the TB-DNA assay. Bone lesion biopsy was performed in 318 patients, with pathology indicating epithelioid granuloma with or without multinucleated giant cell reaction and necrosis. Moreover, tissue Xpert testing revealed Mtb DNA positivity in 69.5% (444/639) of the patients, while 59.6% (371/623) tested positive for tissue bacillus antibodies. Additionally, mycobacterial solid culture yielded a positive result in 38.2% (187/490) of cases, and acid-fast staining was positive in 14.4% (51/356) of the patients’ tissues (**Table 3**, **Figure 3**).

**Table 3:** Comparison with TB culture as a reference standard for various testing data.

Diagnostic techniques		Culture positive	Culture negative	Total	Positive detection rate
T-SPOT.TB	positive	128	69	197	75.8%
	negative	17	129	146	
	Total	145	198	343	
Xpert	positive	141	19	160	69.5%
	negative	13	172	185	
	Total	154	191	345	
TB-DNA	positive	50	16	66	68.5%
	negative	10	36	46	
	Total	60	42	102	
TB-Ab	positive	93	57	150	59.6%
	negative	41	153	194	
	Total	134	210	344	
AFB	positive	39	9	48	14.4%
	negative	79	132	211	
	Total	118	141	259	

The diagnostic sensitivity of T-SPOT.TB was 88.3% (95%CI: 81.9%-93.0%), with a specificity of 65.2% (95%CI: 58.6%-71.5%) and a Youden index of 0.54. TB-DNA demonstrated a sensitivity of 83.3% (95%CI: 71.7%-91.0%) a specificity of 69.2% (95%CI: 55.6%-80.3%), and a Youden index of 0.53. GeneXpert MTB/RIF exhibited a detection sensitivity of 91.6% (95%CI: 86.3%-95.0%), a specificity of 90.1% (95%CI: 85.5%-93.6%), and a Youden index of 0.82. TB-Ab had a sensitivity of 69.4% (95%CI: 61.2%-76.6%), a specificity of 72.9% (95%CI: 66.5%-78.4%), and a Youden index of 0.42. AFB smear microscopy showed a sensitivity of 33.1% (95%CI: 25.2%-42.0%), a specificity of 93.6% (95%CI: 88.3%-96.6%), and a Youden index of 0.27 (**Table 4**).

**Table 4:** Evaluation indicators of each examination technology.

	Sensitivity (95% CI)	Specificity (95% CI)	PPV (95% CI)	NPV (95% CI)	YI value
T-SPOT.TB^a^	88.3% (81.9%-93.0%)	65.2% (58.6%-71.5%)	65.0% (58.2%-71.4%)	88.4% (82.1%-93.0%)	0.54
Xpert ^a^	91.6% (86.3%-95.0%)	90.1% (85.5%-93.6%)	88.1% (82.5%-92.4%)	93.0% (88.7%-95.8%)	0.82
TB-DNA^a^	83.3% (72.0%-90.7%)	69.2% (55.7%-80.1%)	75.8% (64.2%-84.5%)	78.3% (64.4%-87.7%)	0.53
TB-Ab^a^	69.4% (61.2%-76.6%)	72.9% (66.5%-78.4%)	62.0% (54.0%-69.4%)	78.9% (72.6-84.0%)	0.42
AFB^a^	33.1% (25.2%-42.0%)	93.6% (88.3%-96.6%)	81.3% (68.1%-89.8%)	62.6% (55.9%-68.8%)	0.27

TB-Ab, tuberculosis antibodies; AFB, acid-fast staining; PPV, positive predictive value; NPV, negative predictive value; YI, Youden index (YI); ^a^used Wilson Score method.

### 3.4 Drug-resistant analysis

**Figure 4:**
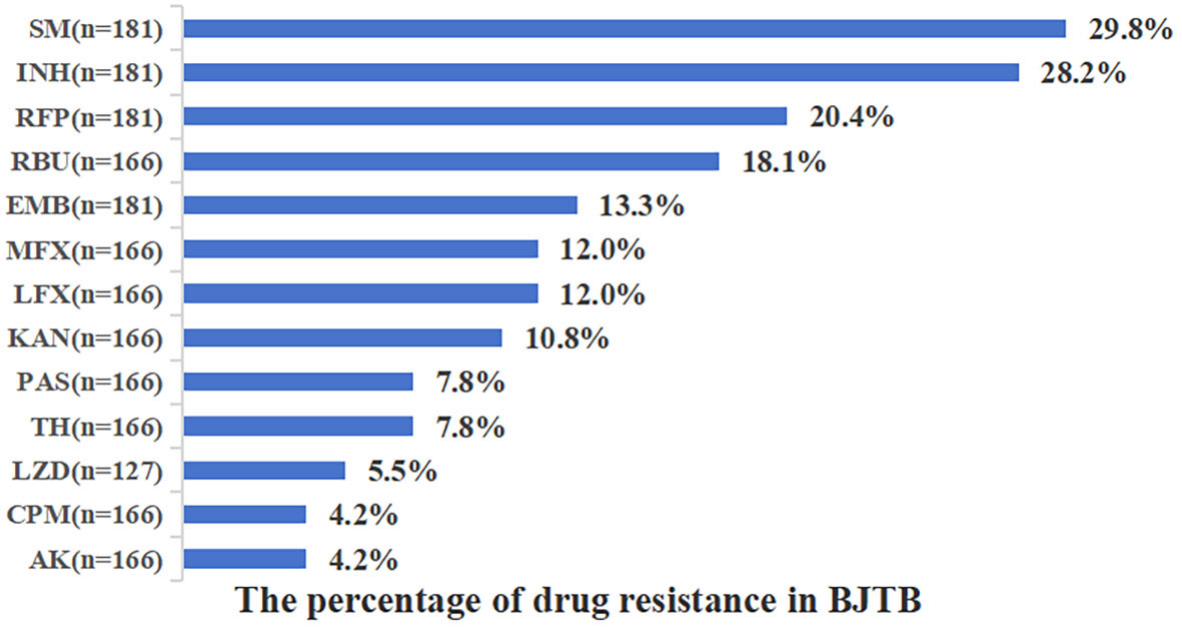
The percentage of drug resistance in BJTB patients.

We evaluated the drug sensitivity of tissue isolates from 181 patients diagnosed with BJTB, with 54 cases of first-line drug resistance (29.8%) and 30 cases of second-line drug resistance (18.2%). Among these isolates, the highest percentage of resistance was observed for Sm at 29.8%, followed by INH at 28.2%, RFP at 20.4%, RBU at 18.1%, EMB at 13.3%, MFX and LFX, both at 12.0%, KAN at 10.8%, TH and PAS, each at 7.8%, LZD at 5.5%. The least resistance was found against AK and CPM, both showing a resistance rate of 4.2% (**Figure 4**). There was no statistically significant difference in drug resistance between patients with spinal TB and those with joint TB (P> 0.05). Among the analyzed Mtb strains (**Table 5**), Rifampicin-resistant tuberculosis (RR-TB) accounted for 20.4% (95% CI: 15.1%-27.0%), and Multidrug-resistant tuberculosis (MDR-TB) accounted for 8.3% (95% CI: 5.1%–13.2%). Pre-extensive drug-resistant tuberculosis (Pre-XDR-TB) constituted 5.4% (95% CI: 3.3%–9.6%), while extensively drug-resistant tuberculosis (XDR-TB) accounted for 1.6% (95% CI: 0.2%-5.6%).

**Table 5:** Drug resistance analysis.

Drug resistance type	Number of drug-resistant cases	Total number of cases	Drug resistance rate (95% CI)
MR-TB^a^	54	181	29.8% (23.6-36.9%)
RR-TB^a^	37	181	20.4% (15.2-26.9%)
MDR-TB^a^	15	181	8.3% (5.1-13.2%)
Pre-XDR-TB^a^	9	166	5.4% (2.9-9.9%)
XDR-TB^b^	2	127	1.6% (0.2-5.6%)

^a^used Wilson Score method ^b^used Clopper-Pearson exact method.

### 3.5 Treatment and outcomes

**Figure 5:**
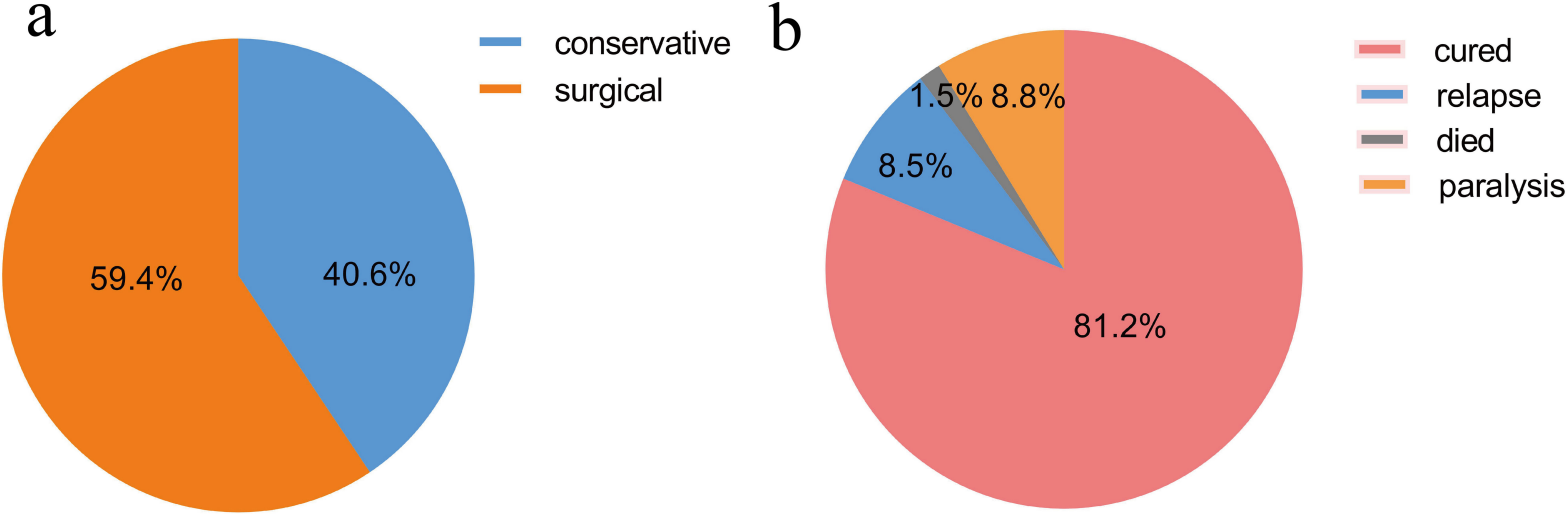
Treatment and outcomes of BJTB cases. **(a)** Treatment of BJTB cases; **(b)** Outcomes of BJTB cases.

Among the 902 patients, 40.6% (366/902) patients were chosen for conservative treatment (quadruple anti-TB agents: isoniazid 0.3 g/d, rifampicin 0.45 g/d, ethambutol 0.75 g/d, and pyrazinamide 0.75 g/d). 59.4% (536/902) of patients underwent surgical treatment after antituberculous chemotherapy 2 weeks later (**Figure 5a**). Within the follow-up period, 81.2% (526/648) patients were cured and achieved satisfactory symptom improvement, 8.5% (55/648) patients experienced a relapse, 1.5% (10/648) died, and 8.8% (57/648) suffered from complete or incomplete paralysis (**Figure 5b**). During the treatment cycle, 8.0% (72/902) individuals presented with drug-induced liver injury, 1.7% (15/902) with acute gastrointestinal reaction syndrome, (0.3%) 3/902 with peripheral neuritis, and 0.6% (6/902) with allergic dermatitis.

## 4 Discussion

BJTB is the most common infection of EPTB, which always causes significant functional impairment, and poses a significant challenge in global public health. However, the true incidence of global BJTB remains unclear due to several factors. These include variations in diagnostic capabilities across different regions, underreporting in certain areas, and difficulty in distinguishing BJTB from other diseases with similar symptoms. Moreover, treatment regimens and surgery strategies for BJTB lack uniformity, showing significant heterogeneity (Khanna and Sabharwal, 2019; Ruparel et al., 2022). To some extent, this situation calls for more of our attention. Our study first described the clinical and epidemiological characteristics of BJTB in Henan Province, the most populous province in China. It also evaluated and analyzed diagnostic techniques and drug resistance patterns. The findings are expected to provide guidance for individualized treatment strategies and grassroots promotion in Henan Province, as well as serve as critical references for the prevention and management of BJTB worldwide.

A prior study indicated that female patients had more than twice the likelihood of male patients to develop EPTB (Ong et al., 2004). However, our study failed to identify a significant association between female gender and bone TB. Similar to previous studies, male-to-female ratio among recruited patients was 1.4:1, significantly lower than that of PTB patients (Wang et al., 2014). In addition, the number of patients suffering from bone TB in rural was 73%, which is significantly higher than that in urban areas. This difference can be attributed to the influence of key risk factors impacting male TB patients, such as alcohol consumption, smoking, and heavy workloads, as well as the inequitable distribution of healthcare resources in Henan Province, a large agricultural region. In developed nations, BJTB predominantly affects the native population over 60 years old (Pigrau-Serrallach and Rodríguez-Pardo, 2013). Studies conducted in southwest China and Beijing revealed that the most common age of onset for BJTB was among 20–39 years. In contrast, the population distribution in our province exhibits a bimodal pattern, with peaks occurring in individuals aged 21–30 years and those 51–60 years old. Hazra et al.’s analysis of osteoarticular TB in the South Indian region showed that joint TB was more frequently observed in children (Hazra et al., 2024). However, there was no significant age differences in patients with joint and spinal TB in our study.

Moreover, as a province with a high burden of TB, our research revealed distinct features of BJTB in Henan compared to the southwestern region of China. Specifically, the time interval from symptom onset to diagnosis for patients with BJTB in our province is relatively short, averaging 6.1 months. This duration aligns closely with reports from European studies, which indicate symptom durations ranging from 4 to 7 months (Colmenero et al., 2004; Guillouzouic et al., 2020). In contrast, research conducted in Hunan Province, China, demonstrated an average interval of 16.0 months from symptom onset to diagnosis for spinal TB patients (Liu et al., 2019). These differences may be attributed to variations in the infrastructure and testing capabilities of TB clinics across regions. Additionally, the proportion of osteoarticular TB cases complicated by active PTB varies significantly. For instance, Shi et al.’s study in Chongqing, China, reported that 14.7% of osteoarticular TB cases were accompanied by PTB (Procopie et al., 2017), while Turgut M.’s investigation of 694 spinal TB patients in Turkey found only 2.7% were associated with active PTB (Qian et al., 2018). In this study, 37.0% of patients exhibited active TB in other organs, and 34.4% had active PTB, which is notably higher than previous findings. This discrepancy might stem from the fact that most patients admitted to our hospital undergo thorough etiological examinations and systematic evaluations, facilitating the detection of EPTB foci.

Our study further compared the clinical manifestations between spinal and joint TB patients. Consistent with previous findings, spinal TB exhibited the highest incidence at approximately 77.6% (Panico et al., 2023). Among the spine TB patients, the lumbar spine was the most commonly involved site. Joint TB predominantly affectes weight-bearing areas such as the hip and knee joints, likely due to their rich vascular supply (Karabella et al., 2022). BJTB was typically caused by the reactivation of latent Mtb during bacteremia from primary infection; however, hematogenous dissemination was detected in only 2.2% of our patients, underscoring the necessity of improved submission rate of blood culture for examination for diagnosing TB in Henan province. Besides, patients with spinal TB presented more classical TB symptoms, including low-grade fever, fatigue, weight loss, and night sweats. This may explain why patients with joint TB (7.3 months) experienced longer diagnostic delays compared to those with spinal TB (5.4 months). In our cohort, patients with spinal TB had an average hospital stay of 62.5 days, while those with joint TB averaged 70.3 days. The insidious nature of the infection and delayed diagnosis likely contributed to the high prevalence of neurological complications and prolonged hospitalization.

According to the statistics from the WHO, only 58% of TB patients in China have bacteriological laboratory evidence, which is below the global average (Organization WH, 2024). For the remaining patients, TB diagnosis relies heavily on clinical manifestations and imaging evidence, which lacks sufficient specificity. Although TB culture and acid-fast staining smears are currently the most widely used laboratory diagnostic techniques for TB, our research indicated that the positive detection rates for BJTB using these methods are only 29.3% and 14.4%, respectively. The low bacterial load in the lesion and the difficulty in obtaining samples have severely limited the ability to detect BJTB cases. Therefore, accurate, rapid, and simple diagnostic methods are essential for the effective management of BJTB. TB-DNA, GeneXpert, and TB-Ab detection demonstrate superior performance compared to traditional culture methods in diagnosing TB and can provide results within two hours. However, there is currently no consensus on adopting these methods as standard diagnostic tools for BJTB (Liu et al., 2021; Biakto et al., 2024), and further research in this area is still deemed necessary. Xpert MTB/RIF is a recently developed nucleic acid amplification test (NAAT) that enables the simultaneous detection of Mtb and RIF resistance within two hours by targeting the *rpoB* gene (Rv0664). This assay has been endorsed by the WHO as the recommended method for the rapid diagnosis of TB (Khan et al., 2024). In our study, a total of 715 specimens were analyzed. Using positive culture as the gold standard, the results demonstrated that Xpert MTB/RIF achieved a comprehensive sensitivity of 91.6% and a comprehensive specificity of 90.1% when diagnosing osteoarticular TB in lesion tissues. These findings highlight its superior sensitivity and specificity for the rapid diagnosis of BJTB, offering valuable insights for clinical decision-making and demonstrating significant potential for promotion at the grassroots level. T-SPOT.TB represents a promising screening tool for BJTB. In our study, the positive rate (78.5%) and sensitivity (88.3%) of T-SPOT.TB were higher than those reported in previous studies (66.3% and 71.4%, respectively) (Li Z. et al., 2023). Nevertheless, due to its relatively low specificity (65.2%), T-SPOT.TB should only be regarded as an important predictor rather than a definitive diagnostic criterion for BJTB.

The control of MDR-TB remains one of the most critical and challenging aspects of global TB prevention and control efforts. However, there is currently a notable lack of research data on drug-resistant BJTB, particularly in developing countries. In this study, 181 Mtb strains were isolated from 902 patients with BJTB for *in vitro* drug susceptibility testing. The overall drug resistance rate was 29.8%, the multidrug resistance (MDR-TB) rate was 8.3%, and the extensively drug-resistant (XDR-TB) rate was 1.6%. Although the MDR-TB rate among patients with BJTB (8.3%) was lower than that observed in PTB cases in the Henan region during the same period (17.3%), it remained substantially higher compared to BJTB patients in South Africa (4%) (Held et al., 2017; Li et al., 2022). These rates were also higher than those reported in previous surveys of BJTB in southwestern China [29.8% vs. 29.0%, 8.3% vs. 7.1%, 1.6% vs. 1.2% (Fang et al., 2024). The primary factors contributing to the high drug resistance rate in patients with BJTB include overuse of rifampin in empirical regimens prior to 2020 and delayed diagnosis due to limited access to medical resources in rural counties. The high MDR rate may reflect ropB S531L and Kat S315T1 mutations prevalence in Henan province (Zhang et al., 2011). Additionally, unlike the drug resistance patterns observed in bone TB cases in India and southwestern China (Qian et al., 2018), in our province, Sm resistance was the highest among first-line drugs at 29.8%, followed by INH (28.2%) and RIF (20.4%). Among second-line drugs, RBU resistance was the most common (18.1%), followed by LFX and MFX (both 12.0%), while AK and CPM showed the lowest resistance rates (both 4.2%) (Yang et al., 2020; Wang et al., 2023). These findings are largely consistent with the drug-resistance characteristics of bone TB reported in Beijing, China. It was worthy to notice that no drug resistance difference was found between spinal TB and joint TB in the present study. Furthermore, the application of molecular diagnostic tools (such as Xpert Ultra, Xpert Omni, TruAT, and genome-wide association studies) to perform in-depth analysis of the molecular profiles of MDR-TB in this region represents an essential direction for future research. Nevertheless, challenges remain, such as the need to improve sensitivity in detecting low-concentration bacterial samples and challenges in economic operation to date (Khan et al., 2024).

## 5 Conclusion

This was the first systematic analysis of the characteristics of patients with BJTB in Henan Province, central China. Special attention should be given to the occurrence of BJTB in men aged 21–30 and over 50, as well as in rural populations. The GeneXpert/RIF assay demonstrated significant diagnostic accuracy for BJTB. In the Central China region, the control of MDR-TB continues to pose a significant challenge in the management of osteoarticular TB. Despite its contributions, this study has certain limitations. As a single-center study, it may introduce bias into the results. Furthermore, the difficulty in obtaining specimens and their low bacterial content could impact the accuracy of drug sensitivity tests. Further studies with multi-center and larger sample sizes are warranted to provide comprehensive insights into prevention and effective control strategies for BJTB in Henan Province and beyond.

## Data Availability

The original contributions presented in the study are included in the article/supplementary material. Further inquiries can be directed to the corresponding author.
